# Dynamic finite-element model for efficient modelling of electric currents in electroporated tissue

**DOI:** 10.1038/srep26409

**Published:** 2016-05-23

**Authors:** J. Langus, M. Kranjc, B. Kos, T. Šuštar, D. Miklavčič

**Affiliations:** 1 C3M d.o.o., Technology park 21, SI-1000 Ljubljana, Slovenia; 2 University of Ljubljana, Faculty of Electrical Engineering, Laboratory of Biocybernetics, Tržaška 25, 1000 Ljubljana, Slovenia

## Abstract

*In silico* experiments (numerical simulations) are a valuable tool for non-invasive research of the influences of tissue properties, electrode placement and electric pulse delivery scenarios in the process of electroporation. The work described in this article was aimed at introducing time dependent effects into a finite element model developed specifically for electroporation. Reference measurements were made *ex vivo* on beef liver samples and experimental data were used both as an initial condition for simulation (applied pulse voltage) and as a reference value for numerical model calibration (measured pulse current). The developed numerical model is able to predict the time evolution of an electric pulse current within a 5% error over a broad range of applied pulse voltages, pulse durations and pulse repetition frequencies. Given the good agreement of the current flowing between the electrodes, we are confident that the results of our numerical model can be used both for detailed *in silico* research of electroporation mechanisms (giving researchers insight into time domain effects) and better treatment planning algorithms, which predict the outcome of treatment based on both spatial and temporal distributions of applied electric pulses.

Electroporation is a biophysical phenomenon in which the permeability of the cell membrane is increased by means of high-amplitude and short duration electric fields^1^,^2^. This allows the introduction or extraction of molecules which otherwise lack transport mechanisms through the cellular membrane. Since the introduction of electroporation, many applications have emerged in medicine^3^, biotechnology^4^ and food processing^5^. The two most prominent medical applications of electroporation for tumour treatment are electrochemotherapy^6^,^7^ and tissue ablation with irreversible electroporation^8^. In electrochemotherapy, electroporation is used to introduce cytotoxic drugs, mainly bleomycin and cis-platin, which have intra-cellular targets and poor membrane permeability. Electroporation thus allows a potentiation of cytotoxic effect to be achieved, of up to a factor of 1000 for bleomycin and 80 for cis-platin^9^,^10^. Irreversible electroporation relies on triggering natural apoptotic and necrotic cell death pathways through extensive cell membrane disruption^11^,^12^,^13^,^14^.

Numerical modelling is a regular approach in the evaluation of electroporation in tissue by predicting the electric current, temperature increase and electric field in the treated tissue due to the application of electric pulses^15^,^16^,^17^,^18^. The major benefit of using numerical simulation (*in-silico* experiment) is elimination of the need for tissue samples (*ex-vivo* experiment) or volunteers (*in-vivo* experiment). It also allows prediction and planning of the outcome of treatment in heterogeneous and complex tissue environments^19^,^20^,^21^,^22^,^23^,^24^. Drawbacks, on the other hand, include the complexity of the simulation domain (different tissue types, complex geometry) and the highly non-linear and time dependent response of the tissue to the applied voltage^25^, which cannot be simulated with present state of the art numerical formulations.

Currently published literature on the effects of electroporation pulses on tissue conductivity is based on either static^26^,^27^ or dynamic conductivity^15^,^16^,^17^,^18^,^19^,^20^,^21^,^22^,^23^,^24^,^25^,^26^,^27^,^28^,^29^ changes. The currently accepted model of electroporation can be summarized in the following equations:


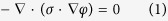



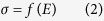


Laplace equation (equation (1)) conserves the electric charge of the system and equation (2) prescribes a functional dependence of tissue conductivity 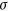 on the local electric field magnitude 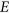. Authors in^25^ report the use of linear, Heaviside and sigmoid models for 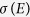. Electric field potential 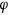 is used for efficiency of numerical calculations and electric field is calculated as a gradient of electric field potential, 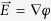.

Equation (1) is a form of charge conservation equation that can be used to describe the state of the electroporated tissue at the end of a long pulse or a train of pulses, when the assumption of a steady state condition within the tissue is reached. This, however, limits the ability to investigate the time course of conductivity changes and electric field distribution during pulse delivery. These models can thus only be validated by voltage and electric current measurements at the end of pulse delivery. Furthermore, it needs to be emphasized that the non-Ohmic behaviour of tissue has been almost exclusively neglected until now.

The aim of our study was therefore to develop a time-dependent numerical model that would replace the currently accepted model of electroporation and provide accurate prediction of the established electric current in the treated tissue throughout the whole duration of applied electric pulses by taking into account tissue capacitance, cell membrane electroporation, as well as relaxation and resealing between the pulses. We believe that the proposed numerical model makes an important step forward in the field of *in silico* experiments of electroporation. It gives researchers in the field a tool for studying time domain effects such as influence of number of pulses (one pulse versus a train of pulses), pulse duration (short versus long pulse), time between the pulses (low versus high pulse repetition rate) in both spatial and temporal domain. We hope that the results of combined spatial and temporal *in silico* experiments of electroporation will eventually lead to improved treatment planning tools.

## Methods

### *Ex vivo* experiment

**Figure 1:**
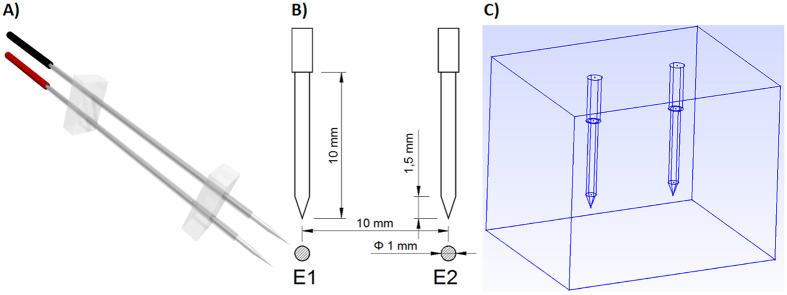
(A) Illustration of the two needle electrodes used in the *ex vivo* experiment, (B) dimensions of exposed part and (C) *in silico* numerical setup. The electrodes were kept 10 mm apart and parallel by two blocks made of acrylic glass. Both electrodes were covered with insulation except at the tip, where 10 mm of the bare electrode was exposed. The electrodes were connected to an electric pulse generator by two connectors (coloured red and black).

Beef liver tissue was obtained from a slaughterhouse that operates in compliance with Slovenian law. Experiments were in agreement with the slaughterhouse since all of their goods are produced strictly for human consumption. The process of slaughtering is regulated by Rules on animal protection and welfare at slaughter (Ur. l. RS, N. 5/2006), which ensures ethical standards of the slaughtering procedure and is in compliance with the European Union Council Directive on the protection of animals at the time of slaughter or killing (93/119/EC). The temperature of the liver tissue was maintained at 4 °C before the beginning of the experiment, when it was allowed to heat up to room temperature. We inserted in the tissue two commercially available needle electrodes (IGEA, Carpi, Italy) with a diameter of 1.2 mm (Fig. 1). The distance between the centres of the electrodes was 10 mm.

As shown in Table 1, nine different sequences of electric pulses were delivered to the tissue. Each sequence contains a train of eight electric pulses that was delivered using a Betatech pulse generator (ELECTRO cell S20, βtech, France). The pulse repetition frequency of 4762 Hz stems from a limitation of the pulse generator that would not allow us to set a spacing of less than 110 μs between the pulses; we will use the terminology of high (4762 Hz) and low (1 Hz) pulse repetition frequency in this article. The current and voltage of electric pulses were measured using a current probe (AP015, LeCroy, USA) and a high-voltage differential voltage probe (ADP305, LeCroy, USA), respectively. Both probes were connected to an oscilloscope (WavePro 7300 A, LeCroy, USA) for acquisition of the measurements. All experiments with the same sequence parameters were repeated five times. The sample was replaced with a fresh one after each electroporation pulse train delivery to ensure identical initial conditions in all electroporation experiments.

**Figure 2:**
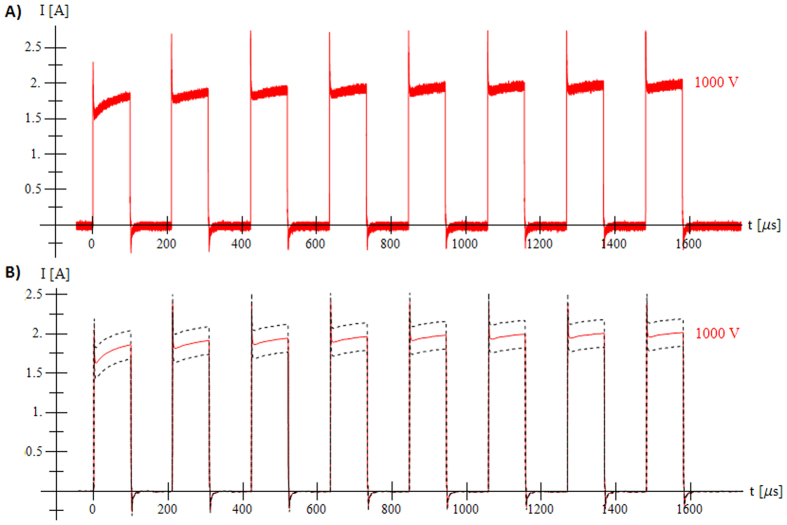
Train of 8 pulses for Sequence 7 (1000 V, short pulse, high pulse repetition frequency). (**A**) Raw experimental data of one measurement and (**B**) down-sampled and averaged value of five experiments (solid line) with standard deviation (dashed line).

While performing experiments with low pulse repetition frequency we were not able to measure the entire 8 second time interval with an acquisition frequency that would allow us to distinguish time dependant effects on the smallest scales (such as the peaks shown in Fig. 2). Data was recorded only during pulse application and so we have no data to compare *ex vivo* and *in silico* experiments during the time between the pulses. High acquisition frequencies (500 MHz for Seq. 1, 4 and 7; 2.5 GHz for Seq. 2, 5 and 8; 250 MHz for Seq. 3, 6 and 9) resulted in a large number of measured points. Each measurement was down-sampled by calculating the average values of applied voltage and measured current for groups of 1000 measured points. For numerical simulations, the average of both voltage and current for all five measurements was calculated for each experiment of the same sequence in order to minimise the effects of tissue non-homogeneity and slight variations between electrode centre-to-centre distances that can occur during needle insertion.

### Numerical model

The currently accepted model of electroporation is sensitive only to applied voltage and cannot distinguish between electroporation scenarios with different number of pulses, pulse duration and pulse repetition frequencies. In order to include these time dependent effects, we developed a numerical model that is defined with the following set of equations:


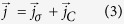



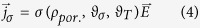



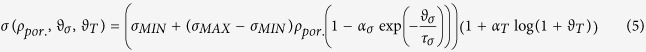



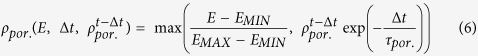



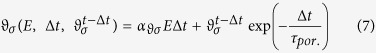



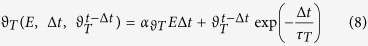



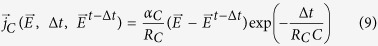


The numerical model in equations 5, 6, 7, 8, 9 includes 14 model parameters, which are listed in Table 2 and discussed below. Current density 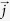 flowing through the tissue is the sum of conductive current density 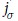 and capacitive current density 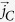. Conductivity *σ* becomes a function of three subsidiary variables: level of poration 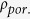, poration damage indicator 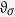 and thermal damage indicator 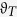. These three variables are functions of the local electric field magnitude 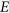, time step 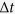 and their own value from the previous time step. The first part of equation (5) is a linear model for the variance of tissue conductivity 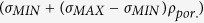 which is supplemented with an additional term 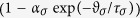 constructed by analysing the shape of the measured current during application of the first pulse. The second term 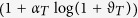 models the rising envelope of measured current with increasing number of applied pulses. The subsidiary variable level of poration 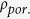 (equation (6)) has a value in the range of [0, 1] and sets an upper limit on local tissue conductivity based on the local electric field magnitude *E*. The subsidiary variables poration damage indicator 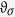 (equation (7)) and thermal damage indicator 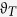 (equation (8)) increase proportionally to the local electric field magnitude during application of the electric pulse and decrease exponentially between the pulses.

Charge conservation equation (equation (1)) of the currently accepted model of electroporation cannot be used as a constitutive equation for our proposed model because of the charge that is stored or released from lipid bilayers within the tissue volume. Our model uses charge conservation equation with charge source/sink term:


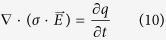


The capacitive current from equation (9) was used to model electric charge source/sink term in our proposed model. Combining equation (9) and equation (10) we write down the proposed constitutive equation:


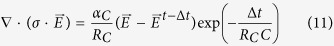


For each time step 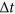 electric field 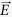 is determined by solving equation (11) using Newton iteration. Convergence criterion for stopping Newton iteration was set to 10^−9^ and solution was typically found in 4 iterative steps, indicating good convergence. Tissue conductivity *σ* was adapted during each iterative step according to equations (5)–(8) and since these equations depend explicitly on electric field 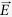 no convergence check for subsidiary variables 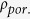, 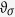 and 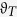 was performed.

The smallest time step 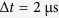 was used when simulating the pulse rise and fall stages (periods of rapid changes in measured current) due to the term 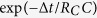 in equation (9). Multiplying 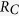 and *C* from Table 2 we see that the RC time constant has a value of 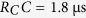 and if 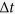 would be much larger than RC time constant, the exponential term would become small and simulation would not reproduce capacitive spikes in simulated current. Simulation time step was as large as 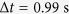  during the time between the pulses in low pulse repetition frequency cases. The adaptive time stepping scheme allowed us to perform the simulation of 8 applied pulses (total duration 7.001 s) with only 190 time steps which is much less than 3.5 × 10^6^ steps it would take to simulate the whole train of pulses using constant time step 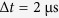.

After the steady state 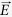 is reached, current densities 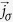 and 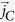 are calculated using equation (4) and equation (9), respectively. Summation of the total current density 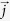 over the surface of one electrode yields the simulated current 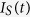 (equation (12)), which is used to compare *in silico* simulation to *ex vivo* experiment.


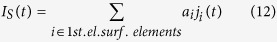



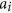
 is the area of the i-th finite element on the electrode surface and 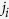 the magnitude of the calculated total current density flowing through the surface of the i-th finite element on the electrode surface.

The set of equations (3)–(11) was transformed into finite element form efficiently using the AceGEN^30^ – symbolic tool for generating finite elements (http://www.wolfram.com/products/applications/acegen/).

Numerical simulation of the *ex vivo* experiment was performed using the finite element method. Two needle electrodes with 1 cm exposed surface were placed 1 cm apart (centre to centre) in a sample of tissue (Fig. 1). Problem geometry was meshed with the GMSH mesher^31^ and the resulting mesh of 98,799 tetrahedral elements imported into AceFEM^32^ package (Wolfram Research, Champaign, IL) in order to perform simulations of electroporation with the developed finite element. A down-sampled and averaged time dependant voltage *U*(*t*) was used as Dirichlet’s boundary condition on the electrode surface for calculating the time dependent electric field and the simulated current *I*_*S*_(*t*) flowing through the electrodes was compared to the down-sampled and averaged time dependent *I*(*t*). Using a measured applied voltage as input proved to be necessary because instant voltage rise and drop in an ideal square pulse produced excessive capacitive response. The simulation domain was closed using Neuman’s type boundary condition on the outer surfaces by prescribing a normal component of current density to 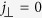.

## Results

Measurements of electric current in *ex vivo* tissue during application of electric pulses are presented in Fig. 2.

**Figure 3:**
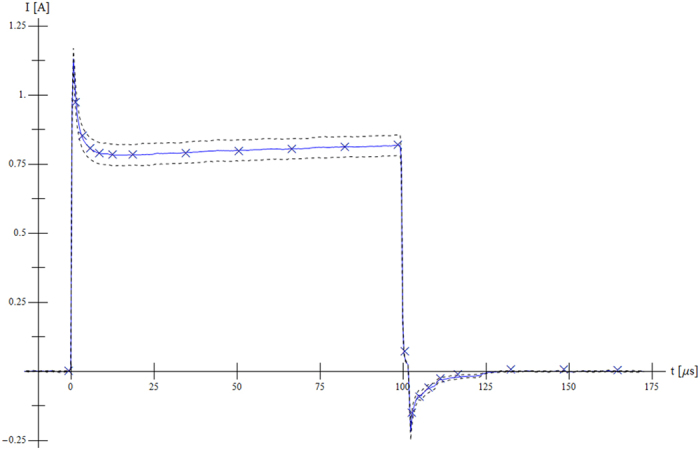
*In silico* experiment fits *ex vivo* measurements. Numerical simulation (“×” marks) of current with respect to measurement (blue solid line) with standard deviation (dashed line) in the case of the first pulse from Sequence 1 (500 V, short pulse, high pulse repetition frequency).

The numerical model was initially tested on the first pulse obtained from all sequences in order to fine tune parameters related to the fast time effects. Figure 3 shows the first pulse from Sequence 1 (500 V, short pulse, high pulse repetition frequency) in which it can be seen that the simulated response (“×” marks) closely follows the measured current (solid line) and captures all transient effects in great detail.

**Figure 4:**
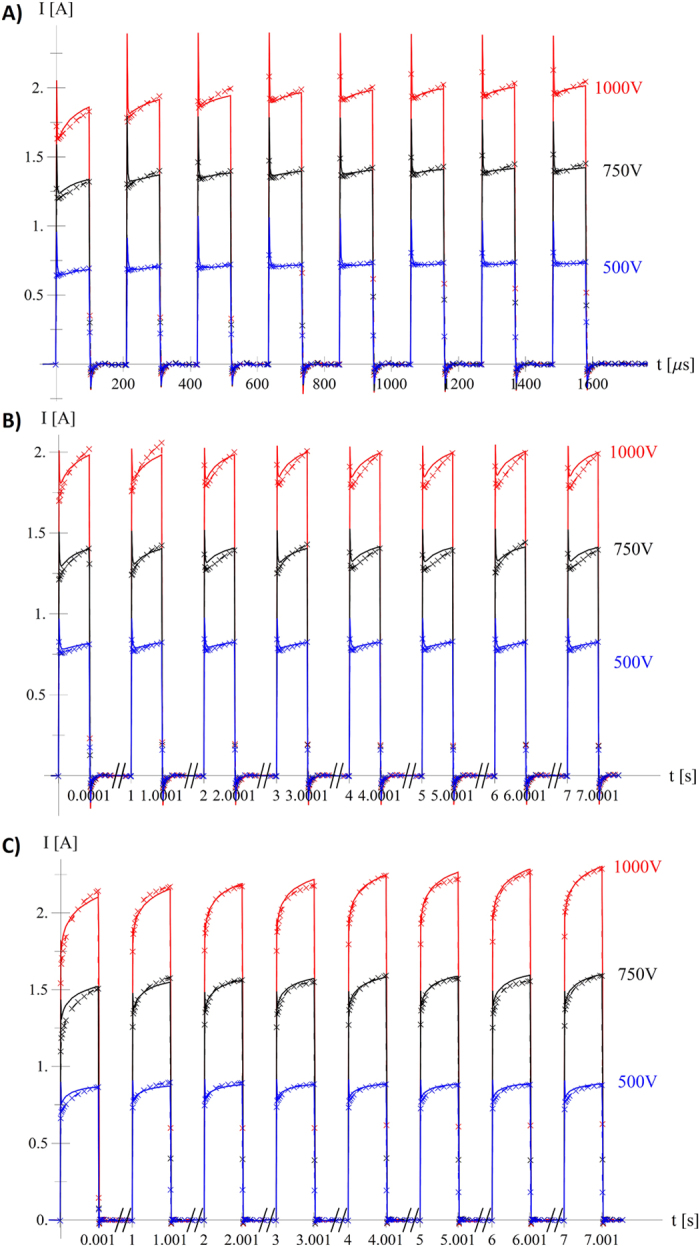
Comparison of measured and simulated current for all nine sequences using optimised *σ*_*MAX*_ for each sequence (Run1). (**A**) Sequences 1 (blue), 4 (black) and 7 (red) – (short pulse, high pulse repetition frequency), (**B**) Sequences 2 (blue), 5 (black) and 8 (red) – (short pulse, low pulse repetition frequency) and (**C**) Sequences 3 (blue), 6 (black) and 9 (red) – (long pulse, low pulse repetition frequency) (bottom), coloured by applied voltage (500 V blue, 750 V black and 1000 V red). Simulation (“×”-marks and dashed line) and measurement (solid line).

The trains of pulses were simulated to test the implementation of time effects on longer time scales. Since the same tissue type was used in all experiments, one would expect that the same material parameters should produce equally good fits for all experiments but this proved to be impossible to achieve. Two batches of parameter values were used in numerical simulations, as shown in Table 3. In the first batch (Run1), parameter *σ*_*MAX*_ was optimised for each sequence while the same values of other parameters were used in all cases. The average value for parameter *σ*_*MAX*_ from Run1 was used in the second batch of simulations (Run2). The results from Run1 are shown in Fig. 4 while an evaluation of the differences between Run1 and Run2 is given in Table 3.

Avg. ΔI was calculated as the average difference between the measured current *I*(*t*) and simulated current *I*_*S*_(*t*) at all simulated points. It can be seen that in Run1, the large number of parameters of the numerical model allows for excellent fit of the simulated results, with an average relative deviation of simulated current (avg. ΔI/I_MAX_) staying below 5% in all cases. Limiting the parameter space in Run2 increases the average deviation ΔI but the 5% mark is exceeded in only three cases.

## Discussion

By observing experimental measurements of current flowing between two electrodes during a train of electroporation pulses (Fig. 2) it is evident that the electrostatic implementation of electroporation finite element cannot reproduce rise of the current during train of short pulses and faster capacitor-like effects during pulse rise and pulse fall time. Several distinct features are observed. We see that the current reaches around 75% of the final amplitude at the first pulse rise and during following pulses follows exponential envelope, effects that we speculate are linked with fast pore creation during pulse rise period and gradual pore growth during the duration of applied electric pulse respectively. During the development of numerical implementation, we observed an inability to accurately simulate the rise of the current amplitude during the train of pulses with only one exponential curve and had to use two rising contributions on two different time scales. We presume that the second contribution 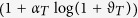 in equation (5), which acts on longer time scales, is due to tissue heating which increases the tissue conductivity^33^,^34^. Observing Seq. 2 (500 V, short pulse, low pulse repetition frequency, see Fig. 4) it can be seen that the measured current reaches repetitive behaviour (all pulses have the same shape and amplitude), indicating the presence of relaxation mechanisms that we believe are connected with pore closing and heat diffusion. Positive and negative current peaks at pulse rise/fall edges are similar to a current charging/discharging capacitor and are believed to be present due to cell membrane capacitance and double layer at the electrode-electrolyte interface.

**Figure 5:**
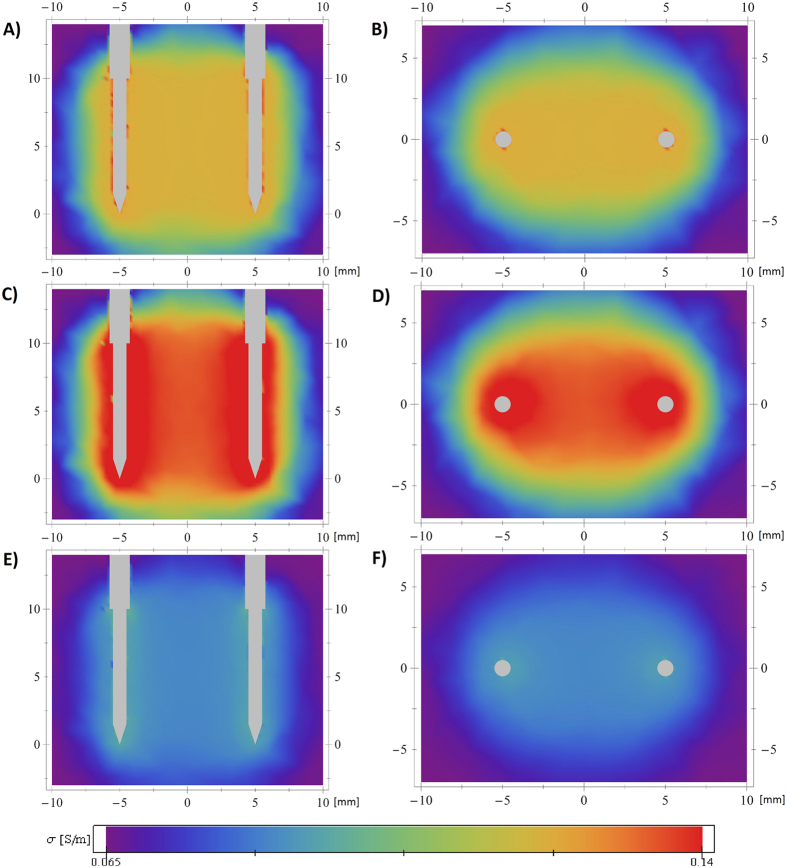
Spatial and temporal evolution of tissue conductivity during application of the first pulse. Sequence 4 (750 V, short pulse, high pulse repetition frequency) and parameters from Run1 were used. Vertical (**A**,**C**,**E**) and horizontal (**B**,**D**,**F**) cross sections over the simulation domain, coloured by calculated tissue conductivity *σ*, at different stages of pulse application. (**A**,**B**) show the beginning of the pulse (when the applied voltage reaches the prescribed value), (**C**,**D**) the end of the pulse and (**E**,**F**) the beginning of the next pulse (maximum relaxation).

**Figure 6:**
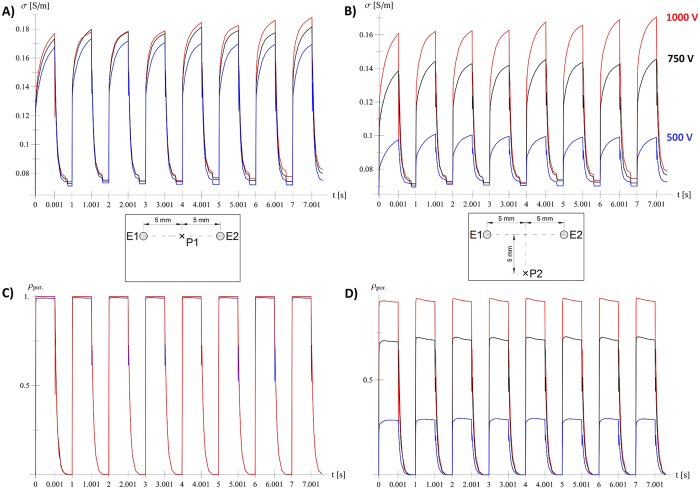
Detailed insight into electroporation process. Time evolution of calculated tissue conductivity *σ* (**A**,**B**) and calculated quantity 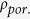 (**C**,**D**) in central point between electrodes (**A**,**C**) and 5 mm from central line (**B**,**D**) for long pulse and low pulse repetition frequency.

We focused on the development of a specialised electroporation finite element by matching measurable macroscopic quantity of the current flowing between the electrodes. The developed and calibrated finite element can be used to extract information that is beyond the reach of experimental methods. One can observe the temporal and spatial evolution of quantities such as electric field strength, current density, calculated tissue conductivity *σ* (Figs 5 and 6), poration indicator 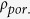 (Fig. 6) etc. at every point and time of the simulated tissue domain.

It can be seen from Fig. 6 that different voltages produce very similar responses at the central point between the electrodes (left images, P1), which indicates that the applied voltage of 500 V is sufficient for successful electroporation of tissue between the electrodes. The calculated values 5 mm from the central line between electrodes (P2), however, show that the response depends strongly on the applied pulse voltage. Figure 6 gives responses for sequences with long pulse duration and low pulse repetition frequency; responses for other sequences can be found in supplementary material online (sequences with short pulse duration and high pulse repetition frequency in Supp. Fig. S1 and sequences with short pulse duration and low pulse repetition frequency in Supp. Fig. S2).

Spatial distributions of the electrostatic field, current density, tissue conductivity etc. have been calculated before^15^,^25^, but our model allows the investigation of both spatial and temporal evolution of the relevant variable (Fig. 5). One possible use of this information would be to calculate the combined time that the tissue conductivity or poration indicator exceeds a (experimentally determined) threshold value and to translate this time into a probability of adequate diffusion of the active substance into porated cells or cell death. Additionally, computing σ as a tensor and E as a vector field would also allow the study of anisotropic tissues, such as muscle or brain tissue, and anisotropy of tissue caused by electroporation^35^,^36^,^37^,^38^.

The amplitude of the current flowing through a pair of electrodes during an electroporation process depends strongly on the electrode placement and tissue geometry, and simulating real-life electroporation experiments involves recreating the geometry in sufficient detail. In order to overcome these problems, a well-defined geometry was chosen for calibration experiments on beef liver samples.

**Figure 7:**
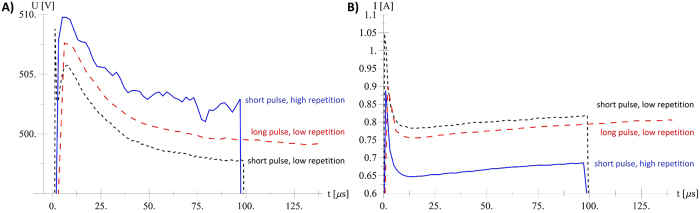
Differences in measured current for the same applied pulse voltage. Comparison of measured applied voltage (**A**) and measured applied current (**B**) for Seq. 1, 2 and 3 (same applied voltage of 500 V, see Table 1).

Because the same tissue type was used in all experiments, a numerical simulation should ideally produce an equally good fit with constant values of model parameters in all cases. It can be seen from Table 3 that while an optimised value of parameter *σ*_*MAX*_ is used for each sequence, deviations in simulated current ΔI are kept low. Differences between experiment and simulation with the same numerical parameters for all cases can partly be ascribed to variations between measurements. When applying the same voltage, the initial response of the current flowing through the tissue should be the same regardless of pulse duration and pulse sequence repetition frequency. Measurements, however, show different behaviour. Figure 7 shows that during the first pulse, the differences between the applied voltages are less than 1% (roughly 5 V/500 V) while measured current at 100 μs flowing between the electrodes varied from 0.68 A to 0.82 A. We suspect the main cause for these current differences is tissue heterogeneity, i.e. biological variability.

The main limitation of our model is that it is empirically based. The system of equations (5)–(9) was constructed using inverse analysis by minimizing the difference between measured electric current 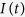 and simulated current 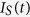. Mechanisms used in the equations are typical for natural phenomena (exponential saturation in 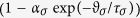, exponential relaxation of auxiliary variables 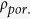, 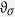 and 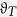) and equal to capacitor charging current in the case of capacitive current density *j*_*C*_. We have neither sound theoretical nor experimental explanation for why these particular equations were chosen for the modelling behaviour of tissue during an electroporation process. The model however can produce accurate prediction of electric current but it is currently limited to particular tissue and tested only on single electrode geometry. Experimental validation on different tissue samples and electrode geometries is needed in order to check whether the proposed set of equations can be applied to arbitrary electroporation scenarios.

By expanding electrostatic implementation with the newly proposed set of equations (3)–(11) we were able to simulate time dependent effects during electroporation treatment in great detail for different combinations of applied pulse voltage, pulse duration and pulse repetition frequency with only one set of parameters (Run2). This achievement gives us confidence that the proposed numerical model is an improvement over the currently accepted model of electroporation and a new step in increasing the validity of *in silico* electroporation modelling.

## Conclusions

Measurements of the current flowing between two electrodes during an electroporation process reveal a complex time dependant rise of current that is non-linearly correlated to applied voltage, number of pulses, pulse duration and pulse repetition frequency. Our goal was to develop an accurate numerical model that would be able to reproduce the measured current with all observed time dependent phenomena.

Electrode geometry and placement have a significant effect on the current amplitude and, with that in mind, we designed an experimental setup that allowed us to position electrodes with great accuracy in the tissue samples. *Ex vivo* measurements of beef liver samples provided reliable data over a broad range of applied pulse voltages, pulse durations and pulse repetition frequency scenarios. Inverse analysis of experimental data led to the development of a numerical model (equations (3)–(11)) and implementation of a specialised finite element that can accurately reproduce measured current when measured voltage is used as the input parameter.

In addition to predicting the current flowing between the electrodes, the developed model can be used to observe spatial and temporal changes of several variables, such as electric field strength, current density, tissue conductivity, poration indicator etc., which can help researchers to gain new insight into the process of electroporation, knowledge that we believe will lead to better understanding of tissue behaviour, electroporation dynamics *in vivo* and improved electroporation treatment planning techniques in the future.

## Additional Information

**How to cite this article**: Langus, J. *et al*. Dynamic finite-element model for efficient modelling of electric currents in electroporated tissue. *Sci. Rep.* **6**, 26409; doi: 10.1038/srep26409 (2016).

## Supplementary Material

Supplementary Information

## Figures and Tables

**Table 1:** Sequences of electric pulses delivered in the tissue.

Sequence number	Peak voltage *U*_e_ [V]	Pulse duration *t* [μs]	Pulse repetition frequency *f* [Hz]
1	500	100	4762
2	500	100	1
3	500	1000	1
4	750	100	4762
5	750	100	1
6	750	1000	1
7	1000	100	4762
8	1000	100	1
9	1000	1000	1

*U*_e_ – peak voltage of electric pulses, *t* – duration of a single pulse, *f* – pulse repetition frequency of a sequence. Eight pulses were delivered in every sequence and each sequence was repeated 5 times, each time on a fresh liver sample.

**Table 2:** Values of model parameters after fitting responses of *in silico* simulation to *ex vivo* measurements.

Symbol	Description	Value (range where applicable)
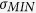	Initial tissue conductivity	0,065 S/m
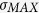	Final tissue conductivity	0,1483 (0,125–0,1675) S/m
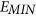	Min electric field magnitude limit in linear conductivity model	20.000 V/m
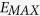	Max electric field magnitude limit in linear conductivity model	40.000 V/m
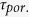	Specific time of poration relaxation	100 μs
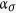	Amplitude of pore growth model	0,35
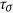	Specific time of pore growth model	15 μs
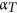	Amplitude of thermal model	0,125
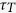	Specific time of thermal diffusion	1,75 s
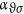	Amplitude of poration damage model	0,0015 m/V
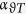	Amplitude of thermal damage model	25,0 m/V
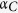	Amplitude of capacitive model	0,0005 m^−2^
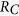	Resistance in capacitive model	15 Ω
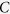	Capacitance in capacitive model	1.2 10^−7 ^F
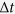	Simulation time step	adaptive from 2 μs to 0.99 s

**Table 3:** Comparison of average deviation of simulated current with respect to measured current for all sequences and two simulation scenarios (Run1– *σ*_*MAX*_ was optimised, other parameters were fixed for all sequences, Run2 – all parameters fixed for all sequences).

Seq. N	I_MAX_ [A]	Run1 – optimised *σ*_*MAX*_		Run2 – same parameters	
		avg. ΔI [A]	avg. ΔI/IMAX	avg. ΔI [A]	avg. ΔI/IMAX
1	0.732	0.036	4.9%	0.058	7.9%
2	0.825	0.031	3.6%	0.059	7.2%
3	0.888	0.033	3.7%	0.043	4.8%
4	1.422	0.043	3.0%	0.059	4.1%
5	1.412	0.039	2.8%	0.072	5.1%
6	1.597	0.050	3.1%	0.062	3.9%
7	2.015	0.045	2.2%	0.047	2.3%
8	2.005	0.041	2.0%	0.090	4.5%
9	2.302	0.063	2.7%	0.094	4.1%
