# Supplementary material for: Dynamic finite-element model for efficient modelling of electric currents in electroporated tissue
Source: Sci Rep. 2016 May 23;6:26409. doi: 10.1038/srep26409 (PMC4876422; doi:10.1038/srep26409)
Supplement: Supplementary Information [file srep26409-s1.pdf]

Dynamic finite-element model for efficient modelling of electric currents in electroporated tissue

J. Langus[1], M. Kranjc[2], B. Kos[2], T. Šuštar[1], D. Miklavčič[2*]

[1] C3M d.o.o., Technology park 21, SI-1000 Ljubljana, Slovenia

[2] University of Ljubljana, Faculty of Electrical Engineering, Laboratory of Biocybernetics (Prof. Damijan Miklavcic, Ph.D.), Tržaška 25, 1000 Ljubljana, Slovenia

Contact: Prof. Damijan Miklavcic, Ph.D.

Tel/fax: +386 1 4768 456 / +386 1 4264 658

email: damijan.miklavcic@fe.uni-lj.si

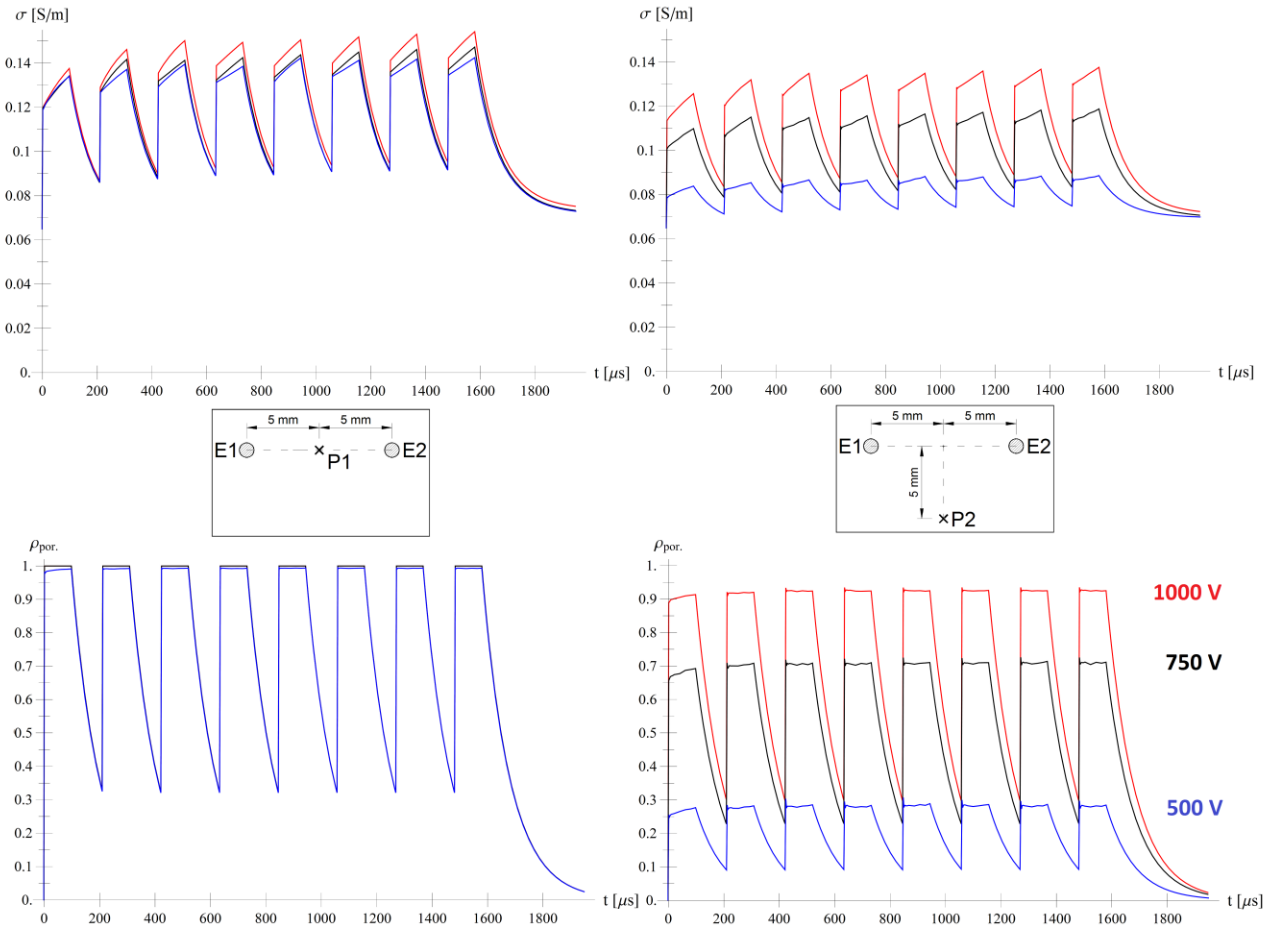

Supplementary Figure S1: Time evolution of calculated tissue conductivity σ (top row) and calculated quantity $\rho_{por.}$ (bottom row) in central point between electrodes (left, P1) and 5 mm from central line (right, P2) for (short pulse duration, high pulse repetition frequency).

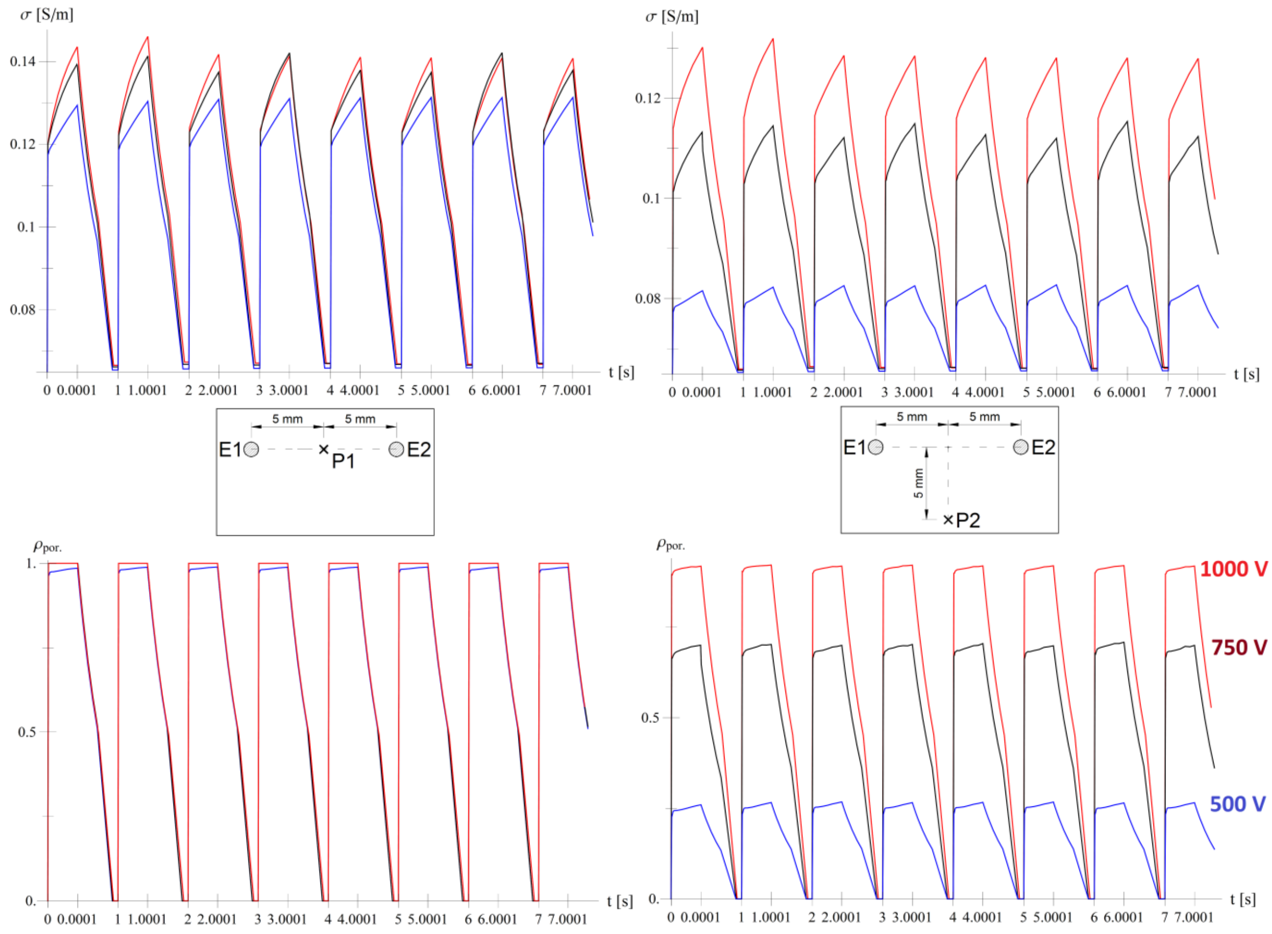

Supplementary Figure S2: Time evolution of calculated tissue conductivity σ (top row) and calculated quantity $\rho_{por.}$ (bottom row) in central point between electrodes (left, P1) and 5 mm from central line (right, P2) for (short pulse duration, low pulse repetition frequency).
